# Comparison of Long-Term Use of Low Dose Rituximab and Mycophenolate Mofetil in Chinese Patients With Neuromyelitis Optica Spectrum Disorder

**DOI:** 10.3389/fneur.2022.891064

**Published:** 2022-05-06

**Authors:** Jie Lin, Binbin Xue, Jia Li, Ruofan Zhu, Juyuan Pan, Zhibo Chen, Xu Zhang, Xiang Li, Junhui Xia

**Affiliations:** ^1^Department of Neurology, The First Affiliated Hospital of Wenzhou Medical University, Wenzhou, China; ^2^Department of Anesthesiology, The First Affiliated Hospital of Wenzhou Medical University, Wenzhou, China

**Keywords:** neuromyelitis optica spectrum disorder, rituximab, mycophenolate mofetil, EDSS, annual relapse rate

## Abstract

**Background:**

Several studies have reported the efficacy and safety of rituximab (RTX) and mycophenolate mofetil (MMF) in neuromyelitis optica spectrum disorder (NMOSD). This study aimed to evaluate the efficacy and safety of long-term use of low-dose RTX and MMF in Chinese patients with NMOSD.

**Methods:**

We retrospectively reviewed data from patients with NMOSD in our hospital. The enrolled patients were administrated different immunosuppressive agents. We accessed annual relapse rate (ARR), neurological disability (Expanded Disability Status Scale, EDSS), time to the next relapse, and adverse events.

**Results:**

EDSS and ARR were both reduced after RTX and MMF treatment. Kaplan-Meier analysis indicated that patients treated with RTX had a longer time to next relapse compared other immunosuppressive agents before RTX (log-rank test: *p* < 0.001). Furthermore, we evaluated the change of EDSS and ARR in RTX and MMF, and patients treated with RTX showed a better reduction. Eleven relapses from seven patients in group RTX and 20 relapses from 14 patients in group MMF were reported during follow-up.

**Conclusion:**

Long-term using of low dose of RTX and MMF were effective and tolerable in Chinese patients with NMOSD. Compared with MMF, RTX showed a better way to reduce the ARR.

## Introduction

Neuromyelitis optica spectrum disorder (NMOSD) (1, 2) is an autoimmune inflammatory disorder of central nervous system (CNS) that attacks areas where water channel protein, aquaporin-4 (AQP4) (3), is distributed. Patients developed severe neurological deficits after accumulated attacks on the optic nerves, spinal cord, brain, and area postrema with the course of relapse-remission.

Previous studies have reported that B cells play an important role in the mechanism of NMOSD; thus, B cell deletion therapy leads to favorable outcomes. Studies have reported that treatment with RTX, a chimera CD20 monoclonal antibody, is an effective preventive therapy for NMOSD patients based on the studies of Cree et al.', as well as in Chinese patients with NMOSD (4–8).

RTX is a monoclonal antibody against CD20 in pre-B and mature B cells. It is used for the prevention and treatment of alloantibody-mediated rejection in transplant recipients, and for the treatment of hematological malignant tumor and rheumatic diseases. Clinical trials have demonstrated that RTX treatment, including long-term RTX treatment, is efficacious, safe, and tolerable (9–13).

The very first report by Cree introduced the regimen (induction: 375 mg/m^2^ per week for 4 consecutive weeks; re-treatment: 1,000 mg/week, 2 weeks apart) as a prevention therapy for patients with NMOSD. The efficiency of RTX have been reported by several clinicians with different regimens; however, there is no consensus on RTX induction and reinfusion. Nonetheless, long-term RTX treatment had been proven to be tolerable and safe in NMOSD patients (10, 11). In our previous study on reduced RTX dosage in Chinese patients with NMOSD, RTX therapy improved the neurological disability and resulted in longer remission compared with non-RTX therapies (7). Studies on reduced RTX dosage for Chinese patients with NMOSD have proven the efficacy (5–7).

MMF suppresses cell-mediated immune and antibody formation by depleting guanosine nucleotides in lymphocytes and inhibiting proliferation (14). Meanwhile MMF decreases production of cytokines, then suppresses recruitment of lymphocytes into inflammatory lesions (15, 16). Previous studies provided evidence that MMF reduced the relapse rate of patients with NMOSD (17, 18).

The study aims to evaluate the efficacy and safety of the long-term use of low-dose Rituximab and MMF in Chinese patients with NMOSD.

## Patients and Methods

### Patients and Treatment Protocol

This study included patients with NMOSD who were treated at the First Affiliated Hospital of Wenzhou Medical University of China between 2013 and 2020. The inclusion criteria included the following: (1) diagnosis of NMOSD following the 2015 revised criteria and (2) at least two infusions of RTX treatment. Patients were infused with RTX at a dosage of 375 mg/m^2^ (body surface area) once during the first treatment. Phenergan and dexamethasone (5 mg i.v.) were applied half an hour before RTX infusion to prevent infusion-related adverse effects. The percentage of CD19+ B cells in peripheral blood lymphocytes was measured before and every 2–3 months after treatment. The same RTX regimen was repeated when the CD19+ B-cell count exceeded 1%. No non-RTX immunosuppressants were administrated with RTX. Tests for immunoglobulin (Ig) A, IgG, and IgM; T lymphocyte spot (T-SPOT) test for tuberculosis infection; tests for fungus; and chest computed tomography were performed before RTX re-treatments. None of the patients were administered concomitant immunosuppressants when initiating RTX treatment.

In the MMF group, all patients received an initial dose of 250 mg twice daily and ratcheted up 500 mg bid based on drug tolerance. Glucocorticoids was tapered after acute attack, and partial patients in this group were treated with a low dosage of glucocorticoids during follow-up.

### Clinical Assessment

A relapse was defined as follows: (1) presentation of new neurological symptoms that were sustained for more than 24 h or original neurological symptoms that deteriorated more than 24 h, and (2) new or enhanced lesions that were evident on T2-weighted magnetic resonance imaging and were related to the symptoms. Intravenous methylprednisolone was administered when patients developed relapsed after treatment. The primary endpoint was the ARR for each patient, and the secondary endpoints were the neurological status, as indicated by EDSS score, and safety. Clinical adverse events (AEs) were recorded throughout the study.

### Statistical Analysis

Statistical analysis was performed using SPSS (version 23.0, IBM, Armonk, NY, USA). The clinical data and demographic of participants, quantitative variables with supposedly normal distribution were described with the mean and standard deviation (SD). The ARR and EDSS before and after treatment were compared via the Mann-Whitney-Wilcoxon matched-pairs signed rank test. Data normality was calculated using the Kolmogorov–Smirnov test. Additionally, Kaplan–Meier analysis was performed to compare the time to next relapse between the RTX and non-RTX treatments. Survival curves were compared using the log-rank test. The COX proportional hazards model for recurrent events was used to compare curves of patients treated with RTX and MMF to obtain the hazard ratios (HR) or odds ratios (OR) and two-sided 95% confidence intervals (CI). In the model, we had adjusted for gender, age of onset, disease duration before initiation to RTX treatment, EDSS score, ARR, previous history of prevention treatments. Statistical significance was set at *p* < 0.05.

## Results

### Patients' Characteristics

Patients who were diagnosed with NMOSD based on the revised criteria of NMOSD in 2015 and treated with RTX and MMF were enrolled. Eighteen patients were treated with RTX and 21 were treated with MMF. In the RTX group, the median age at onset was 27.5 years old (range 15–55 years), and the median time from onset to RTX treatment was 28.5 months (range, 2–150 months). The median follow-up time was 52 months (range, 14–72 months). In the MMF group, the median age at onset and median follow-up time were 36 years (range, 23–56 years) and 42 months (range 8–65 months), respectively. Sero-positivity of AQP4-antibody (AQP4-ab) was detected in 17 and 18 patients in the RTX and MMF group, respectively.

After reviewing the medical records of the group RTX, nine patients received non-steroids immunosuppression (NSIS) before RTX treatment, such as azathioprine (AZA), MMF, cyclophosphamide (CTX), and the remaining patients received glucocorticoids. At the last follow-up, 14 patients continued RTX retreatment, and four patients discontinued treatment because of AEs (*n* = 1), relapses (*n* = 2), personal reason (*n* = 1); the RTX treatment was switched to MMF treatment (*n* = 2). The total number of relapses was 63 before RTX treatment, and 11 acute events in seven patients were reported during the follow-up. Comorbidities, such as Sjogren syndrome (SS) (*n* = 3) and Hashimoto Thyroiditis (HT) (*n* = 1) are showed in Table 1. Resolved hepatitis B was found in 3 patients (HBsAg-negative, HBcAb-positive) in the RTX group.

**Table 1:** Patients' characteristics.

	**RTX**	**MMF**
	**Patients (*n =* 18)**	**Patients (*n =* 21)**
**Gender**		
Female	17	20
Male	1	1
AQP4-ab (positive)	17	18
Age at onset, years, median (range)	27.5 (15–55)	36 (23–56)
Duration before treatment, months, median (range)	28.5 (2–150)	50 (1–145)
Follow-up, months, median (range)	52 (14–72)	30 (8–65)
Autoimmune comorbidities, no.	4	6
Sjogren syndrome, no.	3	3
SLE, no.	0	2
Hashimoto thyroiditis, no.	1	0
Other, no.	0	1
Previous HBV infection, no.	3	2
Possible TB infection, no	0	0
EDSS reduction, no.	16	13
Interval of RTX treatment, months, median (range)	8 (5–2)	–
RTX treatment, no., median (range)	4.5 (2–7)	–
**Relapse after treatment**		
Patients, no.	7	14
Events, no.	11	20
Discontinued RTX or MMF, no.	4	2
**Reasons for discontinuity**		
Relapse, no.	1	0
Adverse events, no.	3	0
Other, no.	0	1 switch to RTX, 1 withdrawal
**Adverse events**		
Respiratory system infection, no.	7	4
Urinary system infection, no.	3	7
TB infection, no.	3	0
Other infection, no	0	2 (herpes zoster)
**Hypoimmunoglobulimia**		
IgG	1	-
IgA	2	-
IgM	7	-
HBV reactivation, no	0	0

*ARR, annual relapse rate; AQP4, aquaporin 4; EDSS, expanded disability status scale; HBV, hepatitis B virus; Ig, immunoglobulin; RTX, rituximab; TB, tuberculosis*.

In the MMF group, six patients had autoimmune diseases (Sjogren syndrome, *n* = 2; systemic lupus erythematosus, *n* = 3; anticardiolipin antibody syndrome, *n* = 1). After treatment, 20 relapses from 14 patients were observed, and the time of first relapse from MMF conducted ranged 7–33 months. Two patients discontinued MMF treatment. During the follow-up, 14 events of infection were reported (Table 1).

### Efficacy

#### Rituximab

**Figure 1:**
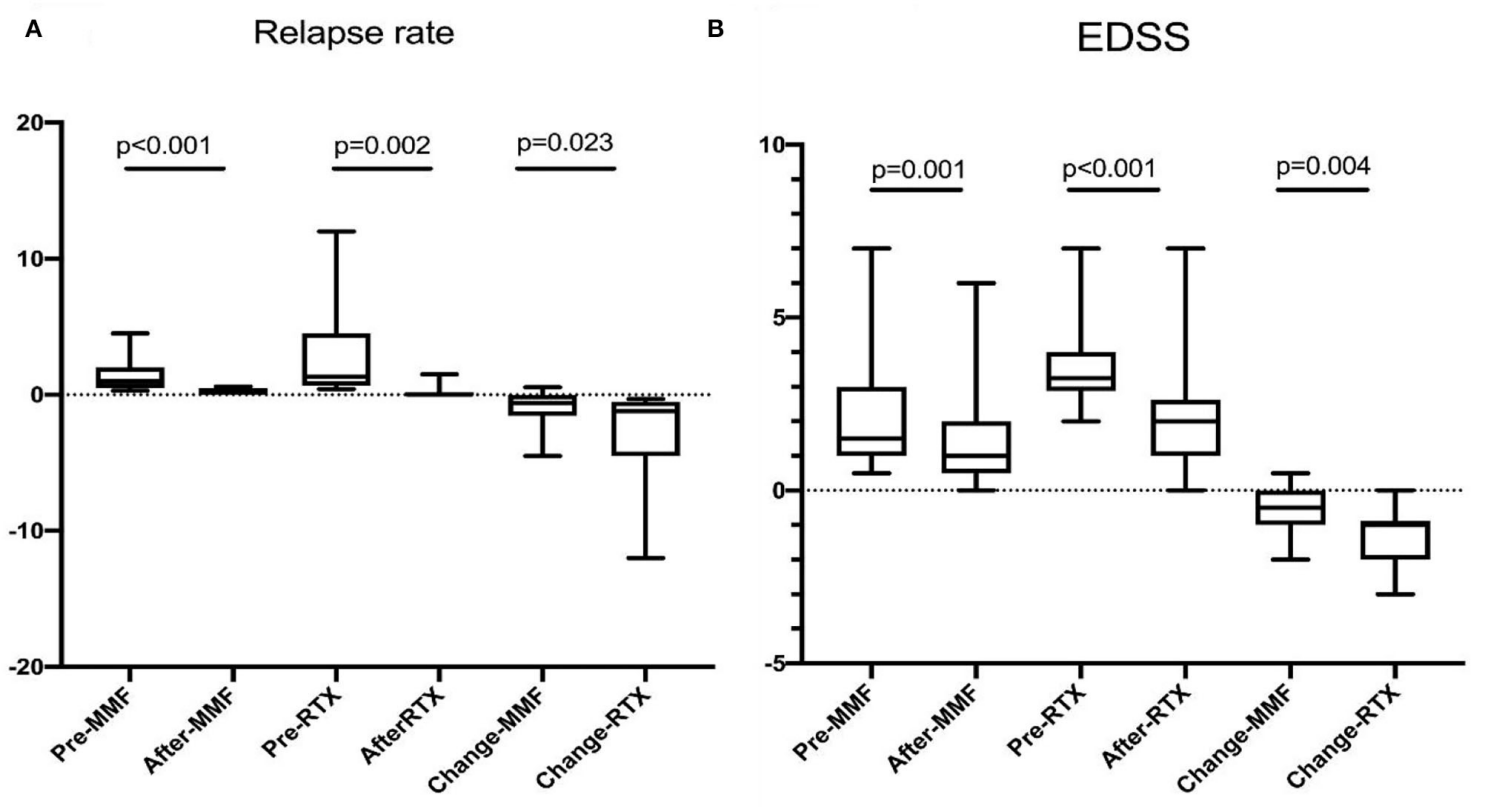
**(A)** Annual relapse rate was reduced from 1.29 ± 1.00 to 0.26 ± 0.23 on MMF (*p* < 0.001), 2.83 ± 3.31 to 0.13 ± 0.35 on RTX (*p* = 0.002). **(B)** EDSS decreased from 2.00 ± 1.49 to 1.45 ± 1.40 on MMF (*p* = 0.001), and 3.39 ± 1.14 to 2.08 ± 1.60 on RTX. Patients treated with RTX had the preferable reduction of ARR (*p* = 0.023) and EDSS (*p* = 0.004).

**Figure 2:**
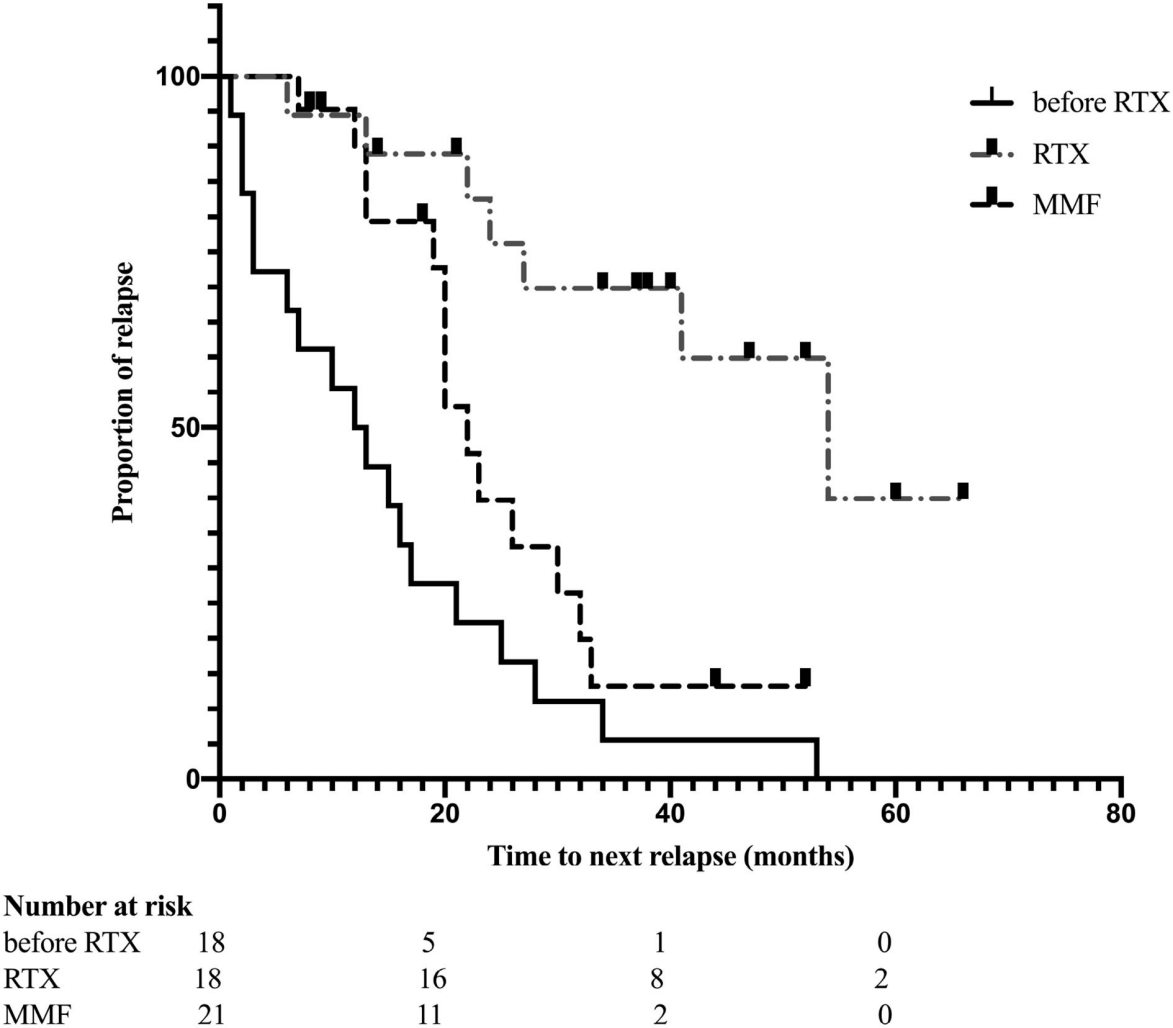
Kaplan-Meier curves estimates the time to next relapse before and after RTX treatment (*p* < 0.001); remission compared between received RTX and MMF treatment (*p* = 0.010).

The median interval of RTX retreatment was 8 months (range, 5–20 months) based on the monitoring of CD19+ B cells. The median number of RTX treatments was 4.5 (range, 2–7). Reduction of ARR was observed in all patients; 11 patients did not experience relapse during RTX treatment. The mean ARR prior to RTX treatment was 2.83 ± 3.31, and the mean ARR after RTX treatment was 0.13 ± 0.35 (*p* = 0.002) (Figure 1). We also evaluated the “time to the next relapse” to assess the treatment efficiency of RTX treatment. Longer remission was observed after RTX treatment compared to other immunosuppression treatment before RTX treatment (log-rank test: *p* < 0.001) (Figure 2). Eleven relapses were reported in seven patients with NMOSD. Four patients relapsed within the first 2 years after RTX treatment.

Furthermore, the neurological disability improved after RTX treatment. Reduction of EDSS was observed in 16 (89%) patients; however, there was no improvement in EDSS in the remaining two patients at the last follow-up compared with those before treatment. The mean (SD) EDSS was reduced from 3.39 (1.14) to 2.08 (1.60) (*p* < 0.001) (Figure 1 and Table 2).

**Table 2:** Efficacy of RTX and MMF.

	**ARR**		**EDSS**		***P^a^***		***P^b^***	
	**RTX**	**MMF**	**RTX**	**MMF**	**ARR**	**EDSS**	**ARR**	**EDSS**
Pre-treatment	2.83 ± 3.31	1.29 ± 1.00	3.39 ± 1.14	2.00 ± 1.49	<0.05	>0.05		
Last follow-up	0.13 ± 0.35	0.26 ± 0.23	2.08 ± 1.60	1.45 ± 1.40			>0.05	>0.05
P^*c*^	0.002	<0.001	<0.001	0.001		

*ARR, annual relapse rate; EDSS, expanded disability status scale; RTX, rituximab; MMF, mycophenolate mofetil.*

*P^a^: Pre-treatment.*

*P^b^: Last follow-up.*

*P^c^: Comparison before and after treatment*.

#### Mycophenolate Mofetil

Reduction of ARR (1.29 ± 1.00 vs. 0.26 ± 0.23) was observed in patients after MMF treatment (Figure 1). Three of twenty-one patients were relapse free, and 16 of 21 had reduced ARR during follow-up. EDSS score was reduced after treatment (2.00 ± 1.49 vs. 1.45 ± 1.40) (Figure 1). Only one patient experienced mild disability progression, and improvement of disability was observed in the remain 12 of 20 patients (Table 2).

ARR and EDSS were compared between the two groups. Compared with the patients in the MMF group, those in the RTX group had higher ARR (*p* < 0.05), and EDSS (*p* > 0.05) prior to treatment, while no significant difference was observed at the last follow-up. Then changes in ARR (ΔARR) and EDSS (ΔEDSS) were introduced to evaluate the efficacy of RTX and MMF. A better reduction of ARR (2.69 ± 3.22 vs. 0.91 ± 1.12, *p* = 0.023) and EDSS (1.31 ± 0.86 vs. 0.55 ± 0.63, *p* = 0.004) was found (Table 3).

**Table 3:** Change of ARR and EDSS.

	**RTX**	**MMF**	***p***
ΔARR	2.69 ± 3.22	0.91 ± 1.12	0.023
ΔEDSS	1.31 ± 0.86	0.55 ± 0.63	0.004

*ΔARR, annual relapse rate change after treatment; ΔEDSS, EDSS change after treatment; RTX, rituximab; MMF, mycophenolate mofetil*.

Kaplan-Meier analysis indicated that patients who received RTX had a longer duration of first relapse after treatment than those in the MMF group (log-rank test: *p* = 0.010) (Figure 2).

**Figure 3:**
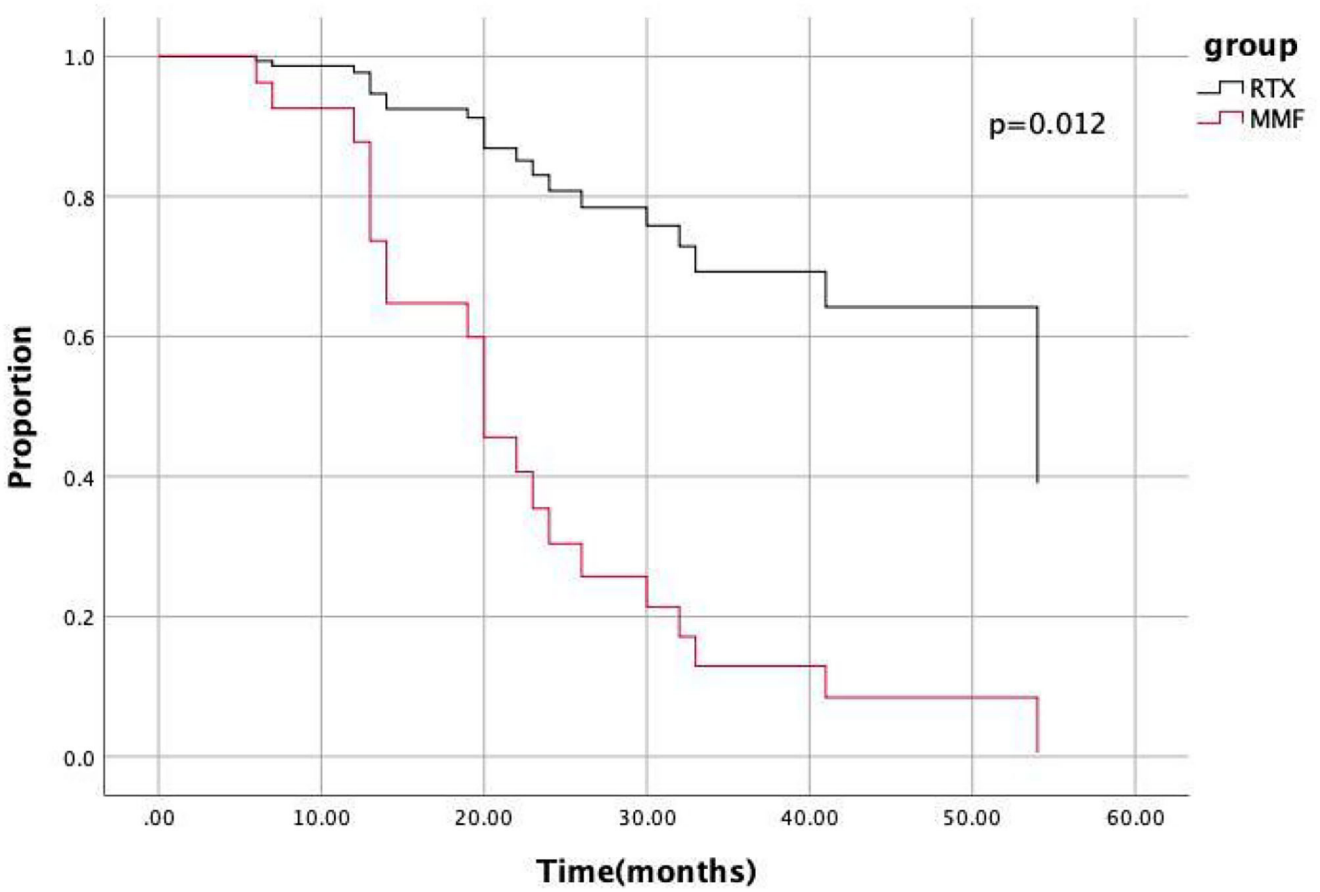
COX hazard model indicated the hazard ratio of the RTX group relative to MMF group was 0.179 (95%CI 0.047–0.0688, *p* = 0.012).

The lower HR of the RTX group relative to the MMF group remained statistically significant (HR: 0.179; 95% CI: 0.047–0.688; *p* = 0.012) after adjusting for variables using the COX hazard model (Figure 3).

No serious AEs, including death, progressive multifocal leukoencephalopathy (PML), or malignancy were reported during follow-up in our study. The most common AE was infection in the RTX group, including respiratory system infection (*n* = 7), and urinary system infection (*n* = 3). Three patients were considered to have possible tuberculosis (TB) based on the T-SPOT, while without clinical symptoms. Of these three patients, one decided to continue RTX treatment with anti-TB treatment, one was switched to MMF treatment after finishing anti-TB therapy, and one discontinued immunosuppressive treatment. No active TB infection was reported during the follow-up period. The patient discontinued RTX reinfusion because of possible TB. Furthermore, hypoimmunoglobulinemia was observed in our patients (IgA: *n* = 1; IgG: *n* = 2; IgG: *n* = 7). During the infusion period, transient hypotension, dizziness, and chest discomfort were reported by the patients. These infusion-related AEs were minor, and no interventions were administered.

Three patients had a history of hepatitis B virus (HBV) infection based on the hepatitis B test; no replication of HBV DNA was found before and after RTX treatment. Seropositivity for hepatitis B core antibody (HBcAb) was observed in two patients, and seropositivity for HBcAb and hepatitis B e antibody (HBeAb) was confirmed in one patient; hepatitis B surface antigen (HBsAg) was negative in all three patients. Thus, they received entecavir (an antiviral drug) along with RTX treatment. No active hepatitis or HBV reactivation (HBVr) was reported during the follow-up period.

Similarly, infection was the most common AE in group MMF (respiratory system infection, *n* = 4; urinary system infection, *n* = 2; herpes zoster, *n* = 2) in five patients. No active hepatitis or HBVr was reported in three patients with previous HBV infection during the follow-up.

## Discussion

The efficiency, safety, and tolerability of RTX treatment have been reported by several studies with long term follow-up (9–13). Cree et al. (4) reported that RTX treatment improved pyramidal, sensory, visual, and bowel/bladder deficits. In their study, patients received intravenous infusions of RTX dosed at a rate of 375 mg/m^2^ for 4 consecutive weeks. RTX retreatment consisted of two intravenous infusions of 1,000 mg administered 2 weeks apart when CD19 B cells were detected. Moreover, neurologists have focused on RTX treatment for NMOSD with different regimens and monitoring indices worldwide (4–8, 10, 11). However, the cost of RTX treatment is high, and many Chinese patients cannot afford it.

A study on reduced RTX dosage was reported by Yang et al. (5). The protocol includED 100-mg infusion once a week for 3 consecutive weeks, followed by additional infusion of the same dosage depending on the repopulation of circulating B-cell. The participants were relapse-free and had reduced EDSS, however, the study included a small sample size and had a short follow-up period. Since then, the efficiency and safety of reduced RTX dosage in Chinese patients have been supported by several, large-scale and longer follow-up studies.

In our study, a remarkable reduction in the ARR and EDSS was observed in RTX and MMF groups, patients treated with RTX seems had the better improvement. However, 11 relapses were reported in seven patients, four of which occurred in the first 2 years after RTX treatment. One patient did not follow the schedule protocol. Furthermore, treatment in one refractory patient switched to treatment with AZA because of relapse 6 months after RTX treatment; however, this patient still suffered three acute events after AZA treatment. This patient still suffered a relapse 6 months after re-initiation of RTX in January 2020. Collongues et al. (19) reported that the relapse rate in refractory NMO patients was 47.7%, and a better treatment for refractory these patients is needed. A relapse rate of 38.9% was calculated in our study, which was not less effective than that described in previous studies (*p* > 0.05) (4–8, 10, 11, 13, 19).

A previous study indicated that RTX reduces ARR and EDSS compared with other immunosuppressive drugs, including MMF. In the present study, patients who received low doses of RTX and MMF also had a significantly reduction in relapse rate and improvement of neurological disability, and those who received RTX showed better improvement. After long-term follow-up, mild infections were recorded in RTX and MMF groups in our study. However, no serious and fatal AE have been reported. The most common AE was infection. Publications (17, 18) also reported that ~4.8–36% of NMO/NMOSD patients received MMF suffered adverse events, including recurrent infection. Hence, safety of long-term using of RTX and MMF was acceptable. A total of 36 studies indicated that the incidence of RTX-associated AEs was not high in patients with NMOSD, and the AEs were commonly mile and moderate. RTX is mostly safer than other immunosuppressive drugs in patients with NMOSD.

Potential TB infection after RTX treatment was observed in the presents study. Previous studies have not reported RTX-related TB infections in patients with NMOSD. However, RTX-related TB infection has been reported in several studies, including in children and with low-dose RTX treatment (20, 21). Thus, TB screening is necessary before treatment and the interval, especially in areas with a high prevalence rate of TB.

Resolved hepatitis B infection (HBsAg-negative, HBcAb-positive) was observed in 3 patients whose HBV-DNA are negative in our study. Previous studies (22–24) have indicated that there is a high risk of HBVr in patients with resolved hepatitis B infection who are receiving immunosuppressant therapy including RTX. Furthermore, studies have indicated antiviral prophylaxis reduces the risk of HBVr (25). Moreover, several studies have recommended that a longer duration (12 months) of antiviral therapy is likely necessary for patients receiving RTX-based therapy (26–28). Screening for HBsAg, HBcAb, and HBV DNA before RTX treatment has been recommended by the National Comprehensive Cancer Network (NCCN), the Centers for Disease Control and Prevention (CDC).

Despite the efficiency and safety of RTX and MMF, financial burdens remain a significant factor in choosing the treatment. A cost of 2,700–3,300 USD per year is necessary based on our RTX regimen and 1,500–3,000 USD per year based on our MMF regimen. Neurological disability was also accumulated after serial attacks, which may lead to incapacity. Therefore, a more effective treatment to disturb the disease progression seems more valuable.

This retrospective study has several limitations. First, our study was based on medical extraction, and small sample size, such as EDSS may lead bias. Second, the washout period to eliminate the influence of prior treatments was absent. Lastly, dynamic tests of APQ4-ab was not available for all patients. However, due to the rarity of NMOSD, limitations are expected. Therefore, a randomized prospective trial is required.

## Conclusion

Long-term using of low dose of RTX and MMF were effective and tolerable in Chinese patients with NMOSD. Compared with MMF, RTX showed a better way to reduce the ARR.

## Data Availability Statement

The original contributions presented in the study are included in the article/supplementary material, further inquiries can be directed to the corresponding authors.

## Ethics Statement

The study involving human participants were reviewed and approved by the Ethics Committee of the First Affiliated Hospital of Wenzhou Medical University. Written informed consent for participation was not required for this study in accordance with the national legislation and the institutional requirements.

## Author Contributions

JLin and BX data collection and drafted the manuscript. JLi, JP, RZ, and ZC made suggestions for improvement. XZ, XL, and JX designed the study and revised the manuscript. All authors have read and approved the version of the manuscript for publication.

## Funding

This study was foundered by the Wenzhou Municipal Science and Technology Bureau (Y20190110 and Y20190135) and the First Affiliated Hospital of Wenzhou Medical University (FHY2019040).

## Conflict of Interest

The authors declare that the research was conducted in the absence of any commercial or financial relationships that could be construed as a potential conflict of interest.

## Publisher's Note

All claims expressed in this article are solely those of the authors and do not necessarily represent those of their affiliated organizations, or those of the publisher, the editors and the reviewers. Any product that may be evaluated in this article, or claim that may be made by its manufacturer, is not guaranteed or endorsed by the publisher.
